# On the Automaticity of the Evaluative Priming Effect in the Valent/Non-Valent Categorization Task

**DOI:** 10.1371/journal.pone.0121564

**Published:** 2015-03-24

**Authors:** Adriaan Spruyt, Helen Tibboel

**Affiliations:** Department of Experimental-Clinical and Health Psychology, Ghent University, Ghent, Belgium; Centre de Neuroscience Cognitive, FRANCE

## Abstract

It has previously been argued (a) that automatic evaluative stimulus processing is critically dependent upon feature-specific attention allocation and (b) that evaluative priming effects can arise in the absence of dimensional overlap between the prime set and the response set. In line with both claims, research conducted at our lab revealed that the evaluative priming effect replicates in the valent/non-valent categorization task. This research was criticized, however, because non-automatic, strategic processes may have contributed to the emergence of this effect. We now report the results of a replication study in which the operation of non-automatic, strategic processes was controlled for. A clear-cut evaluative priming effect emerged, thus supporting initial claims concerning feature-specific attention allocation and dimensional overlap.

## Introduction

In a number of recent publications, Spruyt and colleagues have argued that automatic stimulus evaluation occurs only under conditions that promote selective attention for the evaluative stimulus dimension [1–4]. In line with this assertion, they were able to demonstrate that Feature-Specific Attention Allocation (hereafter referred to as *FSAA*) exerts a strong influence on various markers of automatic evaluative stimulus processing, including the evaluative priming effect [1–3], the emotional Stroop effect [5], the dot probe effect [5], and amplitude variations of the P3a (an ERP marker of attention orienting) evoked by unexpected emotional stimuli [6].

Recently, however, Werner and Rothermund [7] published new evaluative priming data that are difficult to reconcile with the idea that automatic stimulus evaluation is critically dependent upon FSAA. In two statistically powerful experiments, they presented participants with primes and targets that were either positive, negative, or neutral and asked them to categorize the targets as ‘valent’ vs. ‘non-valent’. Despite the fact that selective attention for the evaluative stimulus dimension is required to perform such a task, Werner and Rothermund [7] failed to replicate the evaluative priming effect on the subset of trials (i.e., 25%) that consisted of a valent prime and a valent target (hereafter referred to as *critical evaluative priming trials*).

To account for these null-findings, one might argue that the valent/non-valent categorization task does promote automatic evaluative stimulus processing, but simply fails to pick up the evaluative priming effect because dimensional overlap [8] between the prime set and the response set is missing on the critical evaluative priming trials. In the standard version of the evaluative priming paradigm, both the prime set and the target set consist of stimuli that are either positive or negative and participants are asked to evaluate these stimuli as fast as possible. The prime set is thus related to the response set and several researchers have argued accordingly that Stroop-like response interference is the driving force behind the evaluative priming effect [9–11]. In contrast, in valent/non-valent categorization task, direct response priming can be ruled out as a source of the evaluative priming effect as participants always respond with the same response on the critical evaluative priming trials (i.e., ‘valent’). If it is assumed that response competition is the only mechanism that can produce the evaluative priming effect, the null-findings reported by Werner and Rothermund [7] are anything but surprising.

More recently, however, it has become clear that the evaluative priming effect also replicates in response tasks that rule out dimensional overlap between the prime set and the response set. Spruyt et al. [12], for example, demonstrated that the evaluative match between a prime picture and a target picture can affect target performance even when participants are merely instructed to name the target pictures, an effect that was later replicated by Wentura and Frings [13] and Schmitz and Wentura [14]. Similar effects have also been reported by several other researchers [1–3,15–20]. It therefore seems unwarranted to explain the null-findings reported Werner and Rothermund [7] in terms of the mechanisms that may or may not translate the outcome of the prime-evaluation process into an observable evaluative priming effect [21]. Instead, the findings of Werner and Rothermund [7] seem to suggest that selective attention for the evaluative stimulus dimension is not a sufficient precondition for automatic stimulus evaluation to take place.

Recently, however, Spruyt [4] reported data of two experiments showing clear-cut evaluative priming effects in the valent/non-valent categorization task. The question thus arises how these divergent findings can be accounted for. As a first possibility, Rothermund and Werner [22] argued that the operation of non-automatic matching strategies [23] was not sufficiently controlled for in the studies by Spruyt [4]. Indeed, whereas both the prime set and the target set included valent (i.e., 50%) and non-valent (i.e., 50%) stimuli in the original studies by Werner and Rothermund [7], all the primes were either positive (i.e., 50%) or negative (i.e., 50%) in the studies by Spruyt [4]. As a result, the response ‘valent’ was required whenever the prime and the target were related whereas the same response was needed in just 1/3 of the trials in the absence of an evaluative match (i.e., a neutral target following a valent prime or a valent target following an incongruent prime). It could thus be hypothesized that participants were biased to respond with the response ‘non-valent’ on the incongruent evaluative priming trials. Overcoming such a response bias takes time, thereby (potentially) producing an artifactual evaluative priming effect.

As an alternative explanation, however, it might also be argued that the divergent findings obtained with the valent/non-valent categorization task resulted from the use of different priming parameters [4]. As an example, consider the inter-trial interval (ITI) used in both sets of studies. Whereas Werner and Rothermund [7] used an ITI of just 250 ms, the ITI typically used in the evaluative priming studies by Spruyt and colleagues was (about) 1,000 ms. This procedural difference is potentially important as the valent/non-valent categorization task, when taking into account all trials, is characterized by dimensional overlap between the prime set and the response set. More specifically, neutral primes are compatible with the response ‘non-valent’ but incompatible with the response ‘valent’ whereas positive and negative primes are compatible with the response ‘valent’ but incompatible with the response ‘non-valent’. Participants can thus be expected to perform better on trials consisting of two response-compatible stimuli (e.g., the negative prime word ‘murderer’ followed by the positive target word ‘pretty’) as compared to trials consisting of two response-incompatible stimuli (e.g., the neutral prime word ‘passenger’ followed by the positive target word ‘pretty’). In fact, Werner and Rothermund [7] confirmed that such a response priming effect exerted a (very) strong influence on target performance in both their studies (*d* = 1.82 and *d* = 1.66 in Experiments 1 and 2, respectively). So, whilst the fact that direct response priming can be ruled out as a source of the evaluative priming effect itself, there are good reasons to assume that target responding in the valent/non-valent categorization task is influenced by processes operating at the response selection stage. It has been demonstrated, for example, that response priming effects are typically larger on trials following a response-incompatible trial than on trials following a response-compatible trial [24], a phenomenon also known as the Gratton effect [25]. Likewise, it has been demonstrated that target responding is generally slower on trials following a response-incompatible trial as compared to trials following a response-compatible trial [26], especially when the correct response on trial *t* is related to the task-irrelevant stimulus presented on trial *t*—1, a phenomenon known as negative priming [27,28]. Finally, target responding is often (but not always) found to be faster on response-repetition trials as compared to response-switch trials [29,30]. In sum, processes operating at the response selection stage may inflate the error variance on the critical evaluative priming trials of the valent/non-valent categorization task, thereby making it more difficult to capture the evaluative priming effect. Crucially, the extent to which target responding is affected by phenomena such as negative priming may be dependent on the time interval between successive trials [4,31–33]. Accordingly, differences in the ITIs that were implemented in the studies by Spruyt [4] and Werner and Rothermund [7] may not have been trivial.

In sum, the divergent findings obtained with the valent/non-valent categorization task can be accounted for in several ways. We therefore decided to run a new experiment in which the essential ingredients of the experiments run by Werner and Rothermund [7] were combined with the typical priming parameters used by Spruyt and colleagues. As recommended by Werner and Rothermund [7,22], both the prime set and the target set consisted of valent (i.e., 50.0%) as well as non-valent stimuli (i.e., 50.0%) and each combination of valent and non-valent primes and targets was presented equally often (i.e., 25.0%). This new experiment was thus perfectly balanced in terms of stimulus-response compatibility (see Table 1). In line with Spruyt [4] and Werner and Rothermund [7], we also ensured that each combination of positive and negative primes and targets was presented equally often (i.e., 25.0%) on the subset of critical evaluative priming trials (i.e., valent primes followed by valent targets). As can be seen in Table 1, however, this design implies that the overall proportion of trials on which the prime and the target have the same valence (i.e., 37.5%) is smaller than the proportion of trials on which the prime and the target have a different valence (i.e., 62.5%). Consequently, as was the case in the studies by Werner and Rothermund [7], the likelihood that the response ‘valent’ was required given the presence of an evaluative *mismatch* (i.e., 60.0%) was larger than the likelihood that the same response was required given the presence of an evaluative *match* (i.e., 33.33%). Conversely, the likelihood of the (correct) response ‘non-valent’ was larger given the presence of an evaluative *match* (i.e., 66.67%) than given the presence of an evaluative *mismatch* (i.e., 40.0%). So, if anything, the operation of non-automatic matching strategies should result in a reversed evaluative priming effect [22]. Should a normal (assimilative) evaluative priming effect nevertheless replicate under these conditions, it would be very difficult to argue that it is driven by non-automatic, strategic processes. Conversely, should the evaluative priming fail to replicate in this experiment (or perhaps even reveal a reversed evaluative priming effect), it would be hard to entertain the hypothesis that the use of different priming parameters was responsible for the divergent findings obtained with the valent/non-valent categorization task.

**Table 1:** Trial frequencies within each cell of the design for each block (80 trials in total).

				Evaluative Relatedness					
	Prime Valence			Eval. Priming Trials		All Trials		Response Compatibility	
*Target Valence*	Neutral	Positive	Negative	Con.	Incon.	Con.	Inc.	Comp.	Incomp.
Valent				10	10	10	30	20	20
Positive	10	5	5						
Negative	10	5	5						
Non-valent	20	10	10	-	-	20	20	20	20

This new experiment is also important for an additional reason. Given the fact that both the prime set and the target set included valent as well as non-valent stimuli, the present experiment allows for an exploratory analysis of the extent to which processes operating at the response selection stage may interfere with the detection of the evaluative priming effect in the valent/non-valent categorization task. More specifically, we examined whether the magnitude of the evaluative priming effect was dependent upon phenomena such as the Gratton effect, post-conflict slowing, negative priming, and/or response repetition facilitation.

## Method

### Ethics statement

This research was approved by the Ethics Committee of the Faculty of Psychology and Educational Sciences of Ghent University. All participants affirmed their willingness to participate by signing an informed consent document.

### Participants

Participants were 78 undergraduates at Ghent University (11 men, 67 women, *M*_age_ = 20.50 years, *SD*_age_ = 3.49 years). Whereas some participants were paid €5 in exchange for their participation (*n* = 32), other participants received course credit (*n* = 46). For one participant, the overall error rate on the critical evaluative priming trials was exceptionally high (i.e., 43.75%) in comparison to the rest of the test sample (*M* = 9.69%, *SD* = 6.05%). The data of this participant were excluded. Note, however, that none of the results reported below were contingent upon inclusion or exclusion of this participant. All participants had normal or corrected-to-normal vision.

### Materials and procedure

The stimulus materials and experimental procedures were identical to those used by Spruyt [4], with the following exceptions. First, all participants completed 4 blocks of 80 trials each (i.e., 320 trials in total). Second, within each block of trials, the design was perfectly balanced in terms of stimulus-response compatibility. Likewise, the proportion of congruent and incongruent trials was balanced within the subset of critical evaluative priming trials and (see Table 1). More specifically, participants were presented with 10 evaluative priming trials with congruent stimuli (5 positive/positive trials and 5 negative/negative trials), 10 evaluative priming trials with incongruent stimuli (5 positive/negative trials and 5 negative/positive trials), 20 trials with a neutral target and a neutral prime, 20 trials with a neutral target and a valent prime (10 positive, 10 negative), and 20 trials with a neutral prime and a valent target (10 positive, 10 negative). Due to a program error, 9 participants were actually presented with less than 320 trials. In four of these cases, the final trial list included 319 trials. In three other cases, the final trial list included 318 trials. Finally, there was one participant who completed 317 trials instead of 320 trials. So, in total, 14 trials were lost (i.e., 0.05% of the intended 24,960 trials). Note however that (a) none of the lost trials were critical evaluative priming trials and (b) none of the reported results were contingent upon the inclusion or exclusion of participants who completed less than 320 trials.

Each trial started with a 500-ms-presentation of a fixation cross. Next, after an inter-stimulus interval of 500 ms, the prime was presented for 200 ms. Finally, 50 ms after the offset of the prime (SOA 250 ms), the target was presented until a response was registered. Incorrect responses were followed by a 2000-ms error message. The ITI varied randomly between 500 ms and 1500 ms, with a mean of about 1000 ms. Participants were asked to press a left key in response to neutral targets and a right key in response to positive and negative targets. Instructions emphasized that it was important to respond as fast as possible.

## Results

### The evaluative priming effect

All data reported in this paper can be retrieved from http://figshare.com/. Mean error rates and mean response latencies on critical evaluative priming trials were analyzed by means of a one-way repeated measures ANOVA (congruent vs. incongruent). Mean response latencies were computed after the exclusion of trials on which an incorrect response (9.69%) or a far-out value (2.74%) was registered. Similar to my earlier work, outliers were defined as values that deviated more than 2.5 standard deviations from the mean of an individual participant in a particular cell of the design.

The response latency data were clearly affected by the evaluative congruence of the prime-target pairs. As can be seen in Table 2, responses were faster on congruent trials as compared to incongruent trials, *F*(1, 76) = 6.42, *p* = .01, *MSE* = 816.72. Numerically, the error rates corroborate this finding (see Table 2), but the effect just missed statistical significance, *F*(1, 76) = 3.06, *p* = .08.

**Table 2:** Mean response latencies (in ms) and error rates (in percentages) as a function of prime valence, target valence, evaluative relatedness, and response compatibility (SDs in parentheses).

				Evaluative Relatedness					
	Prime Valence			Eval. Priming Trials		All Trials		Response Compatibility	
*Target Valence*	Neutral	Positive	Negative	Con.	Incon.	Con.	Inc.	Comp.	Incomp.
Response Latency Data									
Valent				612 (84)	624 (89)	612 (84)	625 (85)	619 (86)	626 (84)
Positive	620 (90)	600 (80)	631 (96)						
Negative	632 (82)	616 (88)	624 (94)						
Non-valent	622 (77)	629 (72)	635 (73)	-	-	622 (77)	632 (71)	622 (77)	632 (71)
Error Data									
Valent				9.0 (6.3)	10.3 (7.6)	9.0 (6.3)	11.3 (6.6)	9.7 (6.0)	12.2 (7.4)
Positive	11.8 (8.4)	9.2 (8.4)	10.8 (8.8)						
Negative	12.7 (8.9)	9.9 (10.2)	8.8 (7.8)						
Non-valent	7.5 (6.3)	7.9 (7.9)	9.1 (8.7)	-	-	7.5 (6.3)	8.5 (7.7)	7.5 (6.3)	8.5 (7.7)

*Note*. For the response latency data, all means were computed after exclusion of outlying values. For each participant, outlier criteria were defined as values that deviated more than 2.5 standard deviations from the superordinate cells of the design (e.g., congruent trials with a valent target). The same individual outlier criteria were then used for the subordinate cells of the design (e.g., trials consisting of a positive prime and a positive target).

It may be noted that different outlier elimination criteria were used in the papers by Werner and Rothermund. Werner and Rothermund [7] excluded all response latencies that were either below a fixed 400-ms threshold or more than 1.5 interquartile ranges above the third quartile of an individual response time distribution. In contrast, Rothermund and Werner [22] excluded response latencies that were more than 3 interquartile ranges above the third quartile of the overall response time distribution and no cutoff was used for the leading edge of the response time distribution(s). Reassuringly, when adopting the outlier elimination criterion used by Werner and Rothermund [7], the evaluative congruency effect was highly reliable, *F*(1, 76) = 5.56, *p* <. 05, *MSE* = 290.95. Likewise, a significant evaluative priming effect was obtained when adopting the outlier criterion used by Rothermund and Werner [22], *F*(1, 76) = 9.93, *p* <. 005, *MSE* = 295.03.

### The response priming effect

Mean error rates and mean response latencies were analyzed by means of a one-way repeated measures ANOVA (compatible vs. incompatible). Mean response latencies were computed after the exclusion of trials on which an incorrect response (9.47%) or a far-out value (2.55%) was registered. Outliers were again defined as values that deviated more than 2.5 standard deviations from the mean of an individual participant in a particular cell of the design (compatible trials vs. incompatible trials).

Replicating earlier findings of Werner and Rothermund [7], the response latency data were dependent upon response compatibility. As can be seen in Table 2, responses were faster on compatible trials as compared to incompatible trials, *F*(1, 76) = 10.90, *p* = .001, *MSE* = 236.17. The error data corroborate this finding, *F*(1, 76) = 9.72, *p* = .003. Participants made less errors on compatible trials as compared to incompatible trials.

### The evaluative priming effect as a function of processes operating at the response selection stage

To examine whether the magnitude of the evaluative priming effect was qualified by processes operating at the response selection stage, we first calculated individual indices of (a) the overall response priming effect, (b) the Gratton effect, (c) the post-conflict slowing effect, (d) the negative priming effect, and (e) the response repetition effect. The *overall response priming effect* was defined as the difference between compatible and incompatible trials. The *Gratton effect* was defined as the difference between the magnitude of the response priming effect observed on trials following an incompatible trial and the magnitude of the response priming effect observed on trials following a compatible trial. The *post-conflict slowing effect* was defined as the difference between mean response latencies observed on trials following a response-incompatible trial and mean response latencies observed on trials following a response-compatible trial. The *negative priming effect* was defined as the difference between mean response latencies observed on response-switch trials following a response-incompatible trial and the mean response latencies observed on response-switch trials following a response-compatible trial. Finally, the *response repetition effect* was defined as the difference between response-switch trials and response-repetition trials. These indices were then correlated with the individual evaluative priming scores, defined as the difference between congruent evaluative priming trials and incongruent evaluative priming trials.

Results showed that the magnitude of the evaluative priming effect was unrelated to the overall response priming effect (*r* = -11, *p* >. 20) and the response repetition effect (*r* = .14, *p* >. 20). The evaluative priming effect did correlate, however, with the extent to which participants slowed down after an incompatible response (*r* = -.35, *p* <. 005), the negative priming effect (*r* = -.44, *p* <. 001), and the Gratton effect (*r* = -.29, *p* <. 05).

## Discussion

The results are clear-cut. Replicating the essential ingredients of the experiments run by Werner and Rothermund [7], we obtained a reliable evaluative priming effect using the valent/non-valent categorization task. This finding is important as the design used in the present experiment ensured that strategic, non-automatic processes were unable to contribute to the emergence of this effect. In fact, there are good reasons to argue that a *reversed* evaluative priming effect should have emerged if target responding were affected by strategic processes [22]. As was the case in the studies by Werner and Rothermund [7], the inclusion of a large number of trials consisting of a neutral and a valent stimulus (i.e., 50% of all trials) resulted in a confound between the evaluative relationship between the primes and the targets and the nature of the required response. More specifically, the likelihood of the (correct) response ‘valent’ was larger given the presence of an evaluative mismatch than given the presence of an evaluative match (see Table 1). Conversely, the likelihood of the (correct) response ‘non-valent’ was larger given the presence of an evaluative match than given the presence of an evaluative mismatch. One might therefore hypothesize that participants may have been inclined to respond with the response ‘valent’ on the incongruent evaluative priming trials and with the response ‘non-valent’ on the congruent evaluative priming trials [22]. If this were the case, however, one would predict a reversed evaluative priming effect, not a standard (assimilative) evaluative priming effect as was observed in the present experiment. We can thus firmly rule out the possibility that strategic, non-automatic processes contributed to the emergence of this effect. Accordingly, the results of the present experiment add further weight to the hypotheses that (a) automatic evaluative stimulus processing is critically dependent upon FSAA and (b) processes other than direct response selection can drive the evaluative priming effect.

The results of the present experiment are also important for a second reason. In an earlier publication, Spruyt [4] suggested that the likelihood of capturing the evaluative priming effect with the valent/non-valent categorization task may be dependent upon the extent to which target responding is affected by processes operating at the response selection stage. In line with this assertion, the present experiment revealed that individual evaluative priming scores were negatively correlated with individual measures of post-conflict slowing, the negative priming effect, as well as the Gratton effect. It might thus be interesting to reanalyze the data reported by Werner and Rothermund [7] with these considerations in mind.

Of course, the observation that the overall evaluative priming effect did reach significance in the present experiment also shows that the distortive effect of processes operating at the response selection stage may not always prevent the detection of the evaluative priming effect in the valent/non-valent categorization task. In that respect, the above reasoning can never fully account for the null-findings reported by Werner and Rothermund [7]. It must be to noted, however, that the overall response priming effect observed in the present experiment (i.e., *d* = .38 and *d* = .35, for the response latency data and error data, respectively) was much smaller as compared to the overall response priming effect reported by Werner and Rothermund [7] (i.e., *d* = 1.82 and *d* = 1.66 in Experiments 1 and 2, respectively). It could thus be hypothesized that the evaluative effect was picked up in the present experiment precisely because the influence of processes operating at the response stage was relatively mild.

One (likely) factor that might account for this pattern of results concerns the priming parameters that were implemented in both sets of studies. Whereas Spruyt and colleagues used an ITI of (about) 1000 ms in all their evaluative priming studies, the ITI used in the studies by Werner and Rothermund [7] was atypically short (i.e., 250 ms). This difference in ITI is potentially important as the impact of response selection on trial *t*—1 upon response selection on trial *t* may increase as the ITI decreases [4,31–33]. Further research would be required, though, to identify the exact parametric conditions under which the evaluative priming effect can be found in the valent/non-valent categorization task. Still, the mere fact that we did obtain this effect under strict automaticity conditions is logically sufficient to reject the general assertion that the evaluative priming effect fails to replicate in the valent/non-valent categorization task [7,22].

It must be emphasized, however, that the present findings are insufficient to draw strong conclusions concerning the precise nature of the processes that are responsible for the translation of the prime-evaluation process into an observable evaluative priming effect [21]. As already pointed out by Spruyt [4], (at least) three mechanisms may or may not be at play in the valent/non-valent categorization task: encoding facilitation [12], automatic affective matching, [9], and/or affective-motivational conflict reduction [34]. So, despite the fact that direct response activation can be ruled out as an underlying mechanism of the evaluative priming effect in the valent/non-valent categorization task, it remains unclear to what extent other mechanisms contribute to the effect.

Whilst this limitation of the valent/non-valent categorization task was already emphasized by Spruyt [4], Rothermund and Werner [22] argued that Spruyt [4] interpreted his findings as evidence for an encoding account of the evaluative priming effect. Clearly, this was a misrepresentation of his views. Accordingly, we would like to reiterate that the occurrence of the evaluative priming effect in the valent/non-valent categorization task is insufficient to make general claims about the mechanism(s) that can be operative in the evaluative priming paradigm. To that end, more research would be needed, in all likelihood using response tasks other than the valent/non-valent categorization task.
